# Determination of Antiviral Mechanism of Centenarian Gut-Derived *Limosilactobacillus fermentum* Against Norovirus

**DOI:** 10.3389/fnut.2022.812623

**Published:** 2022-03-28

**Authors:** Ying Li, Junshan Gao, Liang Xue, Yanyan Shang, Weicheng Cai, Xinqiang Xie, Tong Jiang, Huizhen Chen, Jumei Zhang, Juan Wang, Moutong Chen, Yu Ding, Qingping Wu

**Affiliations:** ^1^Guangdong Provincial Key Laboratory of Microbial Safety and Health, Key Laboratory of Agricultural Microbiomics and Precision Application, State Key Laboratory of Applied Microbiology Southern China, Ministry of Agriculture and Rural Affairs, Institute of Microbiology, Guangdong Academy of Sciences, Guangzhou, China; ^2^College of Food Science, South China Agricultural University, Guangzhou, China; ^3^Department of Food Science and Technology, Institute of Food Safety and Nutrition, Jinan University, Guangzhou, China

**Keywords:** norovirus, *Limosilactobacillus fermentum*, antiviral activity, comparative genomic analysis, γ-aminobutyric acid

## Abstract

Although noroviruses are the causative agents of most non-bacterial foodborne disease outbreaks, effective antivirals are currently unavailable. Certain probiotic strains have been reported as active antivirals for norovirus infections, but their mechanisms have not been fully elucidated. Herein, we examined the antiviral potential of 122 lactic acid bacteria isolates against murine norovirus (MNV), a human norovirus surrogate. A centenarian gut-derived strain, *Limosilactobacillus fermentum* PV22, exhibited the strongest MNV antagonism and reduced the viral titer by 2.23 ± 0.38 (log-value) in 5 min with stable activity at 25°C (*P* < 0.01). Genome mining revealed that its antiviral activity can be attributed to the synthesis of γ-aminobutyric acid, and this finding was experimentally verified. Furthermore, we demonstrated the safety of the isolate and its high intestinal colonization ability. In conclusion, we discovered a centenarian gut-derived *L. fermentum* strain with strong anti-norovirus activity and identified its antiviral metabolite. Our results will offer new solutions for the prevention and treatment of food-related norovirus infections.

## Introduction

Norovirus, previously known as the Norwalk virus, is an RNA virus belonging to the family *Caliciviridae* (1). It causes ~677 million gastroenteritis cases worldwide per annum (2). Most norovirus-associated infections are self-limiting, with symptoms including diarrhea, nausea, and fever that resolve within 2–4 days in healthy adults (3, 4); however, recent evidence has revealed that norovirus infections can persist for years in the elderly, neonates, and immunocompromised individuals, where these chronic infections can even be fatal (5–8). Compared to other foodborne pathogens, norovirus is more transmissible as it is transmitted not only *via* food, but also *via* contaminated water and close contact with infected patients (9–13), making the outbreaks extremely difficult to control. Despite global efforts to control norovirus infections, there are no antivirals or vaccines currently available, and the infections remain self-healing (14). As there are over 48 prevalent genotypes of the norovirus, it is challenging to develop a generic vaccine for the whole population (15, 16). Therefore, antivirals for norovirus are urgently needed to control the disease.

However, antiviral studies on norovirus have been hindered by the inability to propagate human norovirus in cell cultures (17). Although recent breakthroughs have led to the successful culturing of the virus in B cells and human intestinal stem cells (18, 19), norovirus can only be propagated at replication levels that are insufficient for antiviral studies (18–20). Consequently, surrogate viruses from the *Caliciviridae* family have been exploited to screen antiviral candidates against norovirus under *in vitro* and *in vivo* conditions. These surrogates include murine norovirus (MNV), porcine sapovirus, feline calicivirus, and rabbit hemorrhagic disease virus (21). MNV is the predominant surrogate because it is classified into the same genus and can be applied to *in vivo* studies in mice with different genetic backgrounds (22, 23). Indeed, numerous antiviral therapies have been developed against norovirus based on the effects of chemical compounds, such as food and plant extracts, against these surrogate viruses. These therapies may offer promising solutions for human norovirus infections (14, 24–26).

Probiotics are live microorganisms that confer health benefits to the host (27), such as maintaining the microbiome balance in the gut mucosa, ameliorating symptoms of irritable bowel disease, and enhancing immune responses against pathogens (28–30). The most commonly used probiotic microbes are lactic acid bacteria (LAB), which are widely consumed as dietary supplements and through fermented foods (31). There is increasing evidence suggesting that probiotics can help prevent and treat viral infections (32, 33). For instance, clinical trials have demonstrated that certain probiotic strains can reduce the severity of rotavirus gastroenteritis and respiratory virus infections (33, 34). Moreover, several studies have confirmed the antiviral effects of certain LAB strains against norovirus, indicating that probiotics could prevent and treat norovirus infections (25, 26).

Although a series of clinical trials have demonstrated the antiviral effects of LAB strains, the underlying probiotic mechanisms remain largely unclear (32). Studies have found that probiotics may exert antiviral effects by secreting active metabolites to hinder viral adsorption and cell internalization (35), producing antiviral substances, such as organic acids and hydrogen peroxide (28), and stimulating the antiviral immune system (30). However, most of these antiviral mechanisms have been explored in respiratory viruses, whereas little is known about how LAB strains function against gastrointestinal viruses. Therefore, an improved understanding of how probiotics act against norovirus will help in the utilization of these strains to control norovirus infections.

Herein, we investigated the antiviral potential of gut-derived LAB isolates against MNV and explored the possible mechanism operating in the strain exhibiting the highest antiviral activity by genome mining analysis. The findings of this study will help in the identification of a new and effective therapeutic approach for norovirus infections.

## Materials and Methods

### Screening of LAB Strains With Antiviral Activity Against MNV

#### Bacterial Isolation and Phylogenetic Analysis

We examined the antiviral potential of 122 LAB strains, 78 of which were isolated from fecal samples of 12 healthy centenarian volunteers in Guangdong and 44 were isolated from traditional fermented foods in Xinjiang. A flow diagram for the selection and verification of the antiviral LAB is shown in Supplementary Figure 1, and detailed information is presented in Supplementary Table 1.

Genomic DNA from all LAB strains was extracted using a genomic DNA extraction kit (Magen Biotech, Guangzhou, China). LAB species were identified using *16S rRNA* gene sequencing. The *16S rRNA* gene was amplified using universal primers (27F, 5′-AGA GTT TGA TCC TGG CTC AG-3′; 1492R, 5′-ACG GCT ACC TTG TTA CGA CTT-3′) as described by Garrity (36). Polymerase chain reaction products were sent to GENEWIZ (GENEWIZ, Inc., Suzhou, China) for Sanger sequencing. *16S rRNA* gene sequences were compared with reference sequences from GenBank (http://www.ncbi.nlm.nih.gov/BLAST).

#### Cell and Virus Cultures

We used murine macrophage RAW264.7 cells (American Type Culture Collection TIB-71) for antiviral activity assessment and cytotoxicity evaluation, and Caco-2 cells (American Type Culture Collection HTB-37) for determining the cell adhesion activity. Both RAW264.7 and Caco-2 cells were maintained at 37°C in a 5% CO_2_ incubator (Thermo Fisher Scientific, Waltham, MA, USA) in complete Dulbecco's modified Eagle's medium (DMEM; Thermo Fisher Scientific) containing 10% (vol/vol) fetal bovine serum (Gibco, Grand Island, NY, USA) and an antibiotic-antimycotic mixture (100 U penicillin, 100 μg/ml streptomycin, and 0.25 μg/ml amphotericin B; HyClone, Logan, UT, USA).

MNV was propagated by inoculating confluent RAW264.7 cells for 4 h and incubating them in complete DMEM at 37°C and 5% CO_2_ for 48 h. The infected cells were subjected to three freeze/thaw cycles (−80/37°C) and pelleted by centrifugation at 10,000 × *g* and 4°C for 1 min. The virus particles in the supernatant were collected, filtered, and stored at −80°C until further use.

The MNV titer in RAW264.7 cells used the 50% tissue culture infectious dose (TCID_50_) value. Briefly, RAW264.7 cells (10,000 cells per well) were seeded in 96-well plates and incubated in complete DMEM for 24 h at 37°C in a 5% CO_2_ incubator. After incubation with serial ten-fold dilutions of 100 μl of MNV suspensions (8 replicates per dilution) for 48 h, the complete cytopathic effect was observed microscopically. Viral titers were determined and expressed as the TCID_50_ using the Reed–Muench method (37). The MNV titer used in this study was –log TCID_50_ 5.04 ± 0.26.

#### Minimum Non-toxic Dilution Preparation

All LAB strains were grown in de Man Rogosa Sharpe (MRS) broth at 37°C for 48 h in an anaerobic workstation (Don Whitley Scientific, W Yorkshire, UK). The LAB cells were then pelleted by centrifugation at 10,000 × *g*, 4°C for 10 min, and the supernatants were collected using 0.22 μm microfilters. The pH of the cell-free serum (CFS) and uninoculated MRS broth controls was adjusted to 7.35–7.45 using sodium hydroxide (Sigma-Aldrich, St. Louis, MO, USA) and samples were stored at −80°C until further use.

The cytotoxicity of the pH-adjusted CFS samples was assessed using Cell Counting Kit-8 (CCK-8; Glpbio, Montclair, CA, USA). Five-fold dilutions of the CFS were prepared and cytotoxicity was re-evaluated if RAW264.7 cell viability failed to remain at 100% after 24 h of incubation with the CFS. CFS with the lowest toxicity was defined as minimum non-toxic dilutions (MNTDs).

#### High-Throughput Assay to Screen the Antiviral Potential of MNTDs

The viability of RAW264.7 cells after 24 h of co-incubation with MNTDs treated with MNV was used as a high-throughput screening index for the antiviral potential of LAB. Briefly, MNTDs of the 122 LAB strains were added to the same volume of MNV suspensions in DMEM and incubated at 25°C for 30 min. Cell viability was tested using CCK-8 after incubating 100 μl of RAW264.7 cell suspension (10,000 cells per well) with 10 μl of each LAB-treated virus suspension in 96-well plates for 24 h at 37°C in a 5% CO_2_ incubator. pH-adjusted MRS-treated viral suspension was chosen as a positive control, and DMEM-treated viral suspension was chosen as the method control.

### Evaluation of Antiviral Effects of LAB Strains

To evaluate the actual antiviral effects of MNTDs of different LAB isolates, we estimated the titers of the MNTD-treated MNV using TCID_50_ as described in Section Cell and Virus Cultures.

MNTDs from the top ten LAB strains with the strongest antiviral potential were added to the same volume of MNV suspensions (−5.04 ± 0.26 log TCID_50_/mL) in DMEM, incubated at 25°C for 30 min, and MNTD-treated MNV titrations were evaluated. Each treatment was performed in triplicate, with virus suspensions in the same volume of DMEM and pH-adjusted uninoculated MRS broth as the negative controls. Notably, the MNTDs in this experiment were obtained from the LAB CFS after 48 h of fermentation without dilution. We identified a centenarian gut-derived isolate, *Limosilactobacillus fermentum* (*L. fermentum*) PV22, as the strongest antiviral LAB strain and further investigated its potential active metabolites.

### Genomic Mining for the Antiviral Metabolites of *L. fermentum*

#### Genomic DNA Library Preparation

Genomic DNA libraries were constructed using AMT Rapid DNA-Seq Kits for Illumina (CISTRO, Guangzhou, China). Fragmentation, end-repair, adaptor ligation with Illumina adapters, size selection with beads, and library DNA amplification were performed according to the manufacturer's instructions. Libraries were assessed using an Agilent Bioanalyzer 2100 (Agilent Technologies, Santa Clara, CA, USA) and a Qubit 3.0 fluorometer (Invitrogen, Waltham, MA, USA). DNA sequencing was performed on the Illumina Nextseq 550 platform with a High Output v2.5 kit (Illumina, San Diego, CA, USA).

Long reads of microbial genomic DNA libraries were prepared using the Rapid Barcoding Sequencing Kit (Nanopore, Oxford, UK) and were sequenced on the Nanopore MinION platform with R9.4.1 flow cells (Nanopore).

Low-quality reads from Illumina sequencing were filtered and removed using Trimmomatic software (v0.39) (38). Low-quality and short reads from Nanopore sequencing were filtered using Filtlong software (v0.2.0) (https://github.com/rrwick/Filtlong). The filtered Illumina and Nanopore reads were aligned into *de novo* assembled contigs using Unicycler software (v0.4.8) (39).

#### Genome Annotation and Comparative Genome Analysis

Pan-genome analysis of the Prokka output was performed using Roary (v3.11.2) with a BLASTP identity cut-off of 95% (40). The LAB strain core-genome was generated using Harvest software (v1.1.2), with ATCC 14931 as the reference genome (41). Following core-genome alignment, recombination analysis and removal of putative recombined regions were performed using Gubbins (42). Linear sequence comparisons of different *L. fermentum* strain genomes were visualized using Easyfig (v2.2.3) (43).

### Quantification and Verification of Antiviral Metabolites in the LAB Strains

#### Quantification of Antiviral Metabolites in the LAB Strains

By comparative genome mining, a strain-specific gene, *gadB*, was identified as the potential antiviral metabolite of *L. fermentum* PV22. Therefore, quantification of γ-aminobutyric acid (GABA) in MNTDs of LAB strains was performed to validate the result of genome mining. Quantification of GABA was done using AB SCIEX Triple Quad 5500 LC-MS/MS system (Sciex, Framingham, MA, USA) as described by Bottiglieri (44). In brief, MNTDs were diluted 1:1 with a 40% acetonitrile/40% methanol solution and hydrolyzed before being injected into the Triple Quad liquid chromatography-tandem mass spectrometry (LC-MS/MS) system. Each assay was quantified using a five-point standard curve and was linear from 0.63 to 80 μM for total GABA; the quantification of GABA in each MNTD was done using the Analyst software of AB SCIEX Triple Quad 5500 LC-MS/MS system.

#### Verification of Antiviral Metabolites in the LAB Strains

To verify the antiviral effects of GABA on the virus, we compared the MNV titers treated with MNTDs of *L. fermentum* PV22, pH-adjusted MRS with a similar dose (44.67 mg/L) of GABA (Aladdin, Shanghai, China), and pH-adjusted MRS at 25°C for 30 min. We determined the MNV titer using TCID_50_ as described in Section Cell and Virus Cultures.

### Safety Assessment of *L. fermentum*

#### Safety Assessment of the Genomic Level of *L. fermentum* PV22

Protein-coding genes of *L. fermentum* PV22 were annotated using other public databases, including the Virulence Factor Database (VFDB), Comprehensive Antibiotic Research Database (CARD), and Resfinder Database (45–47) for potential risks of virulence factors and antibiotic-resistant genes.

#### Evaluation of the Hemolytic Activity of *L. fermentum* PV22

A single colony of *L. fermentum* PV22 was streaked on 5% sheep blood agar plates and incubated for 48 h at 37°C. Hemolytic activity was detected by the presence of clear zones around the colony. *Staphylococcus aureus* ATCC 25923 was chosen as the positive control.

#### LAB Antibiotic Susceptibility Testing of *L. fermentum* PV22

The susceptibilities of *L. fermentum* PV22 to ampicillin, gentamicin, kanamycin, streptomycin, erythromycin, clindamycin, tetracycline, and chloramphenicol were determined using the broth microdilution method (48). Resistant and susceptible readings were distinguished by the minimum inhibitory concentration cut-off values determined by the European Food Safety Authority (EFSA) (49).

#### Gastrointestinal Tract Tolerance of *L. fermentum* PV22

The tolerance of *L. fermentum* PV22 to the gastrointestinal tract was assessed according to the method described by Pradhan et al. (50). Briefly, 0.35% (w/v) pepsin was dissolved in acidified PBS (pH 3.0) to simulate gastric juice, whereas 1.1% (w/v) NaHCO_3_ and 0.1% (w/v) trypsin were dissolved in PBS (pH 8.0) to simulate pancreatic juice. The LAB was suspended and incubated in simulated gastric juice for 3 h at 37°C before being collected and resuspended in simulated pancreatic juice for 24 h at 37°C. The bacterial survival rate was estimated using the MRS agar plate enumeration method.

#### Cell Adhesion Activity

The cell adhesion activity was assessed using the method described by Jensen et al. (51). Briefly, Caco-2 cells were seeded in a six-well plate, and LAB were added at a multiplicity of infection of 1:100. After 2 h of co-incubation at 37°C, the Caco-2 cells were washed gently with PBS three times to remove the unattached bacteria. Then, the Caco-2 cells were treated with 50 μl trypsin-EDTA (Gibco) for 5 min, and the attached bacteria were collected for serial dilution using the pour-plate technique in MRS agar. The adherence percentage was calculated using the equation:


$$\begin{array}{l} \chi \;(\%)=\frac{N_{2h}}{N_{0h}\;}\times 100 \end{array}$$

### Statistical Analysis

Cell viability tests and MNV titrations were performed in triplicate. Data are presented as the mean ± standard deviation. The comparisons of cell viability and MNV titrations among different groups were performed *via* Wilcoxon rank-sum test using PASW Statistics 18.0.0 (IBM, Armonk, NY, USA). The results with *P* < 0.05 were deemed statistically significant.

## Results

### LAB Taxonomy and Isolation Characteristics

**Figure 1:**
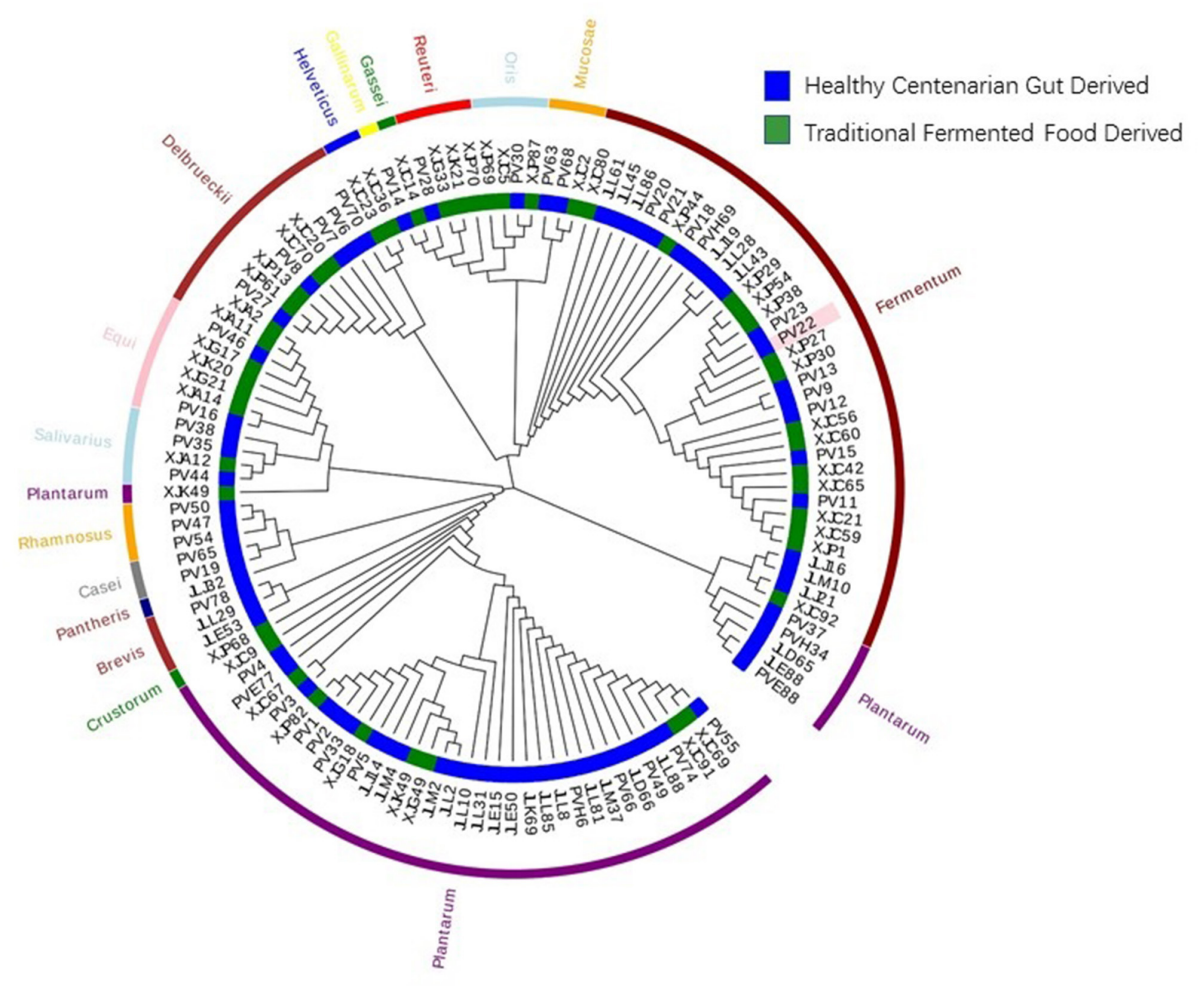
Phylogenetic tree of the 122 strains of lactic acid bacteria (LAB). The phylogenetic tree of 122 LAB strains was constructed based on *16S rRNA* sequences using the neighbor-joining method. The inner circle of the phylogenetic tree indicates the sources from which each LAB strain was isolated. The outer circle of the phylogenetic tree indicates the species of the LAB strains.

The 122 LAB isolates included in this study were derived from 16 different species. The taxonomy and the source of isolation of the LAB strains were analyzed using a *16S rRNA*-based phylogenetic tree (Figure 1). Seventy-eight strains were isolated from healthy centenarians, and the remaining 44 were isolated from traditional fermented food. The taxonomic composition of the LAB strains was as follows: *Lactiplantibacillus plantarum* (*L. plantarum*) 32.79%; *Limosilactobacillus fermentum* (*L. fermentum*) 27.05%; *Lactobacillus delbrueckii* (*L. delbrueckii*) 9.84%; *Ligilactobacillus equi* (*L. equi*) 6.56%; *Limosilactobacillus oris* (*L. oris*) 3.28%; *Limosilactobacillus reuteri* (*L. reuteri*) 3.28%; *Ligilactobacillus salivarius* (*L. salivarius*) 3.28%; *Lacticaseibacillus rhamnosus* (*L. rhamnosus*) 2.46%; *Limosilactobacillus mucosae* (*L. mucosae*) 2.46%; *Levilactobacillus brevis* (*L. brevis*) 2.46%; *Lacticaseibacillus casei* (*L. casei*) 1.64%; *Companilactobacillus crustorum* (*L. crustorum*) 0.82%; *Lactobacillus helveticus* (*L. helveticus*) 0.82%; *Lactobacillus gallinarum* (*L. gallinarum*) 0.82%; *Lactobacillus gasseri* (*L. gasseri*) 0.82%; *Lacticaseibacillus pantheris* (*L. pantheris*) 0.82%.

### Antiviral Potential of MNTDs

**Figure 2:**
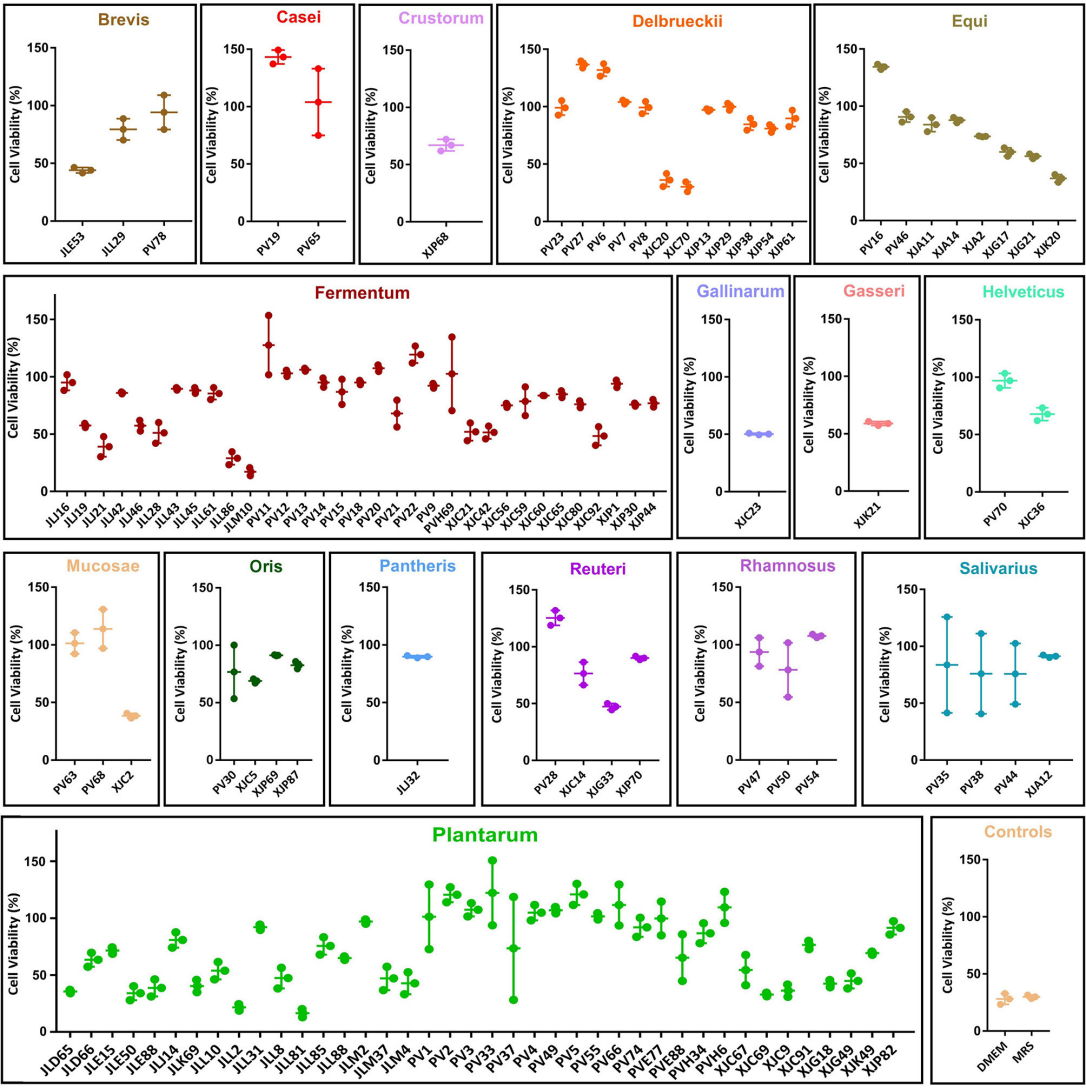
Antiviral potential of the minimum non-toxic dilution (MNTD) of the 122 lactic acid bacteria (LAB) strains. The antiviral potentials were measured by determining RAW264.7 cell viability after 24 h of incubation with MNTD-treated murine norovirus. The antiviral potentials of LAB strains are grouped according to their species. The cell viability was shown in dot and the average cell viability and the standard deviation were shown in bars.

To screen for the antiviral potential of 122 LAB strains, we measured cell viability after 24 h of co-incubation with MNTDs and MNV (Figure 2). Cell viability varied from 16.58 to 143.22% after co-incubation with MNTDs of different LAB strains.

Further analysis revealed that the centenarian gut-derived LAB isolates exhibited a higher average antiviral potential than fermented food-derived isolates (91.40 ± 29.71 vs. 68.04 ± 20.08%, *P* < 0.01). Among the 16 LAB species analyzed, *L. casei* displayed the best antiviral potential, with an average cell viability of 123.60% (*P* < 0.05). The screening showed that 24.59% of the MNTDs could protect RAW264.7 cells from virus-induced apoptosis and helped them maintain cell viability after MNV infection.

### Antiviral Activity of MNTDs

#### Antiviral Activity of MNTDs in the Strains With the Highest Antiviral Potential

**Figure 3:**
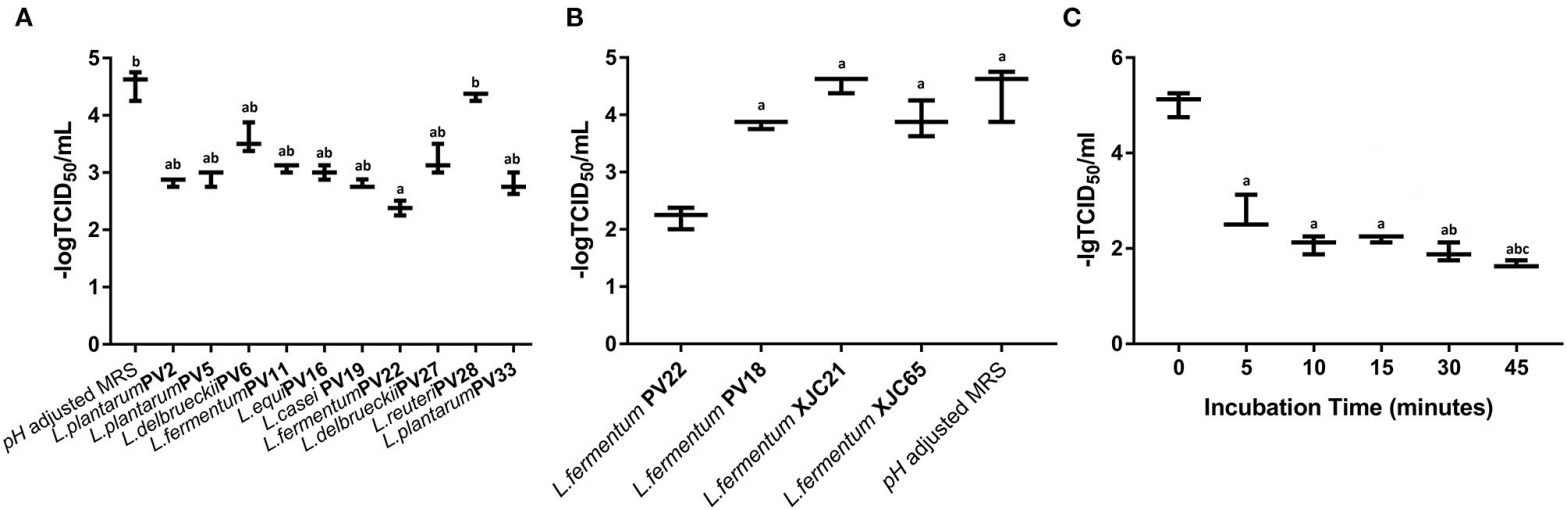
Antiviral activity of the most potential minimum non-toxic dilution (MNTD) obtained from different lactic acid bacteria (LAB) isolates. **(A)** The antiviral activity of the MNTDs from the ten *Limosilactobacillus* strains with the most antiviral potential during a 15 min incubation; pH-adjusted MRS was used as control. a indicates *P* < 0.05 compared to control; b indicates *P* < 0.05 compared to *L. fermentum* PV22. **(B)** The antiviral activity of MNTDs from four different isolated strains of *L. fermentum* during 15 min incubation; pH-adjusted MRS was used as a negative control. a indicates *P* < 0.05 compared to *L. fermentum* PV22. **(C)** The antiviral activity of MNTDs of *L. fermentum* PV22 at different incubation periods. The murine norovirus (MNV) titer determined as 50% tissue culture infectious dose (TCID_50_), shown as dot, with the mean ± standard deviation in bars; a indicates *P* < 0.01 compared to the 0-min group, b indicates *P* < 0.05 compared to the 5-min group whereas c indicates *P* < 0.05 compared to both the 15-min and 30-min groups.

Next, we evaluated the antiviral activities of MNTD of the ten LAB strains with the best antiviral potential against MNV (Figure 3A). The -log TCID_50_ value of different MNTD-treated MNVs was as follows: *L. plantarum* PV2 2.84 ± 0.07, *L. plantarum* PV5 2.92 ± 0.14, *L. delbrueckii* PV6 3.58 ± 0.26, *L. fermentum* PV11 3.08 ± 0.07, *L. equi* PV16 3.00 ± 0.13, *L. casei* PV19 2.79 ± 0.08, *L. fermentum* PV22 2.38 ± 0.13, *L. delbrueckii* PV27 3.21 ± 0.26, *L. reuteri* PV28 4.33 ± 0.07, and *L. plantarum* PV33 2.79 ± 0.19. In particular, the MNTD of *L. fermentum* PV22 exhibited a higher antiviral activity than most of the LAB isolates (*P* < 0.05), with a decrease in MNV activity from 4.54 ± 0.26 to 2.38 ± 0.13 after co-incubation for 15 min at 25°C (*P* < 0.01). Thus, the MNTD of *L. fermentum* PV22 exhibited the highest antiviral effect among all LAB strains.

#### Antiviral Activity of CFS From Four *L. fermentum* Strains

To determine whether the antiviral activity of LAB is related to the taxonomic characteristics, we analyzed the MNTD antiviral activity of three other *L. fermentum* strains: *L. fermentum* PV18 was isolated from fecal samples of healthy centenarians, whereas *L. fermentum* XJC21 and *L. fermentum* XJC65 were isolated from traditional homemade yogurt. MNTDs of *L. fermentum* PV18 and XJC21 reduced MNV toxicity (*P* < 0.05). The MNTD of *L. fermentum* XJC65 showed no antiviral activity, and that of *L. fermentum* PV22 exhibited more significant antiviral activity than that of any other isolated *L. fermentum* strain (Figure 3B; *P* < 0.05). Together, these results suggest that the antiviral activity of PV22 is strain-specific.

#### Antiviral Activity Assessment of *L. fermentum* PV22 Along With Co-incubation Time

To determine the optimum co-incubation time for *L. fermentum* PV22 MNTDs, we evaluated the antiviral effect of MNTDs at various time points. As shown in Figure 3C, we observed that *L. fermentum* PV22 MNTDs significantly reduced the MNV titer after 5 min of incubation and that the antiviral effects increased with co-incubation time for up to 45 min, when –log TCID_50_ decreased from 5.04 ± 0.26 to 1.67 ± 0.07 (*P* < 0.01). This result indicates that the strong antiviral effect of *L. fermentum* PV22 is rapid and persistent.

### Genome Analysis of *L. fermentum* PV22 Antiviral Activity

#### Genomic Features of *L. fermentum* PV22

We sequenced the *L. fermentum* PV22 genome to explore its genomic features. The complete genome comprised one chromosome of 2,190,470 bp with an average GC content of 51.22%. The genome was predicted to contain 2,278 protein-coding genes, 15 rRNAs, and 58 tRNAs. The detailed genomics characteristics of *L. fermentum* PV22 and the other *L. fermentum* isolates are shown in Supplementary Table 2.

#### Comparative Genomic Analyses of *L. fermentum* PV22

**Figure 4:**
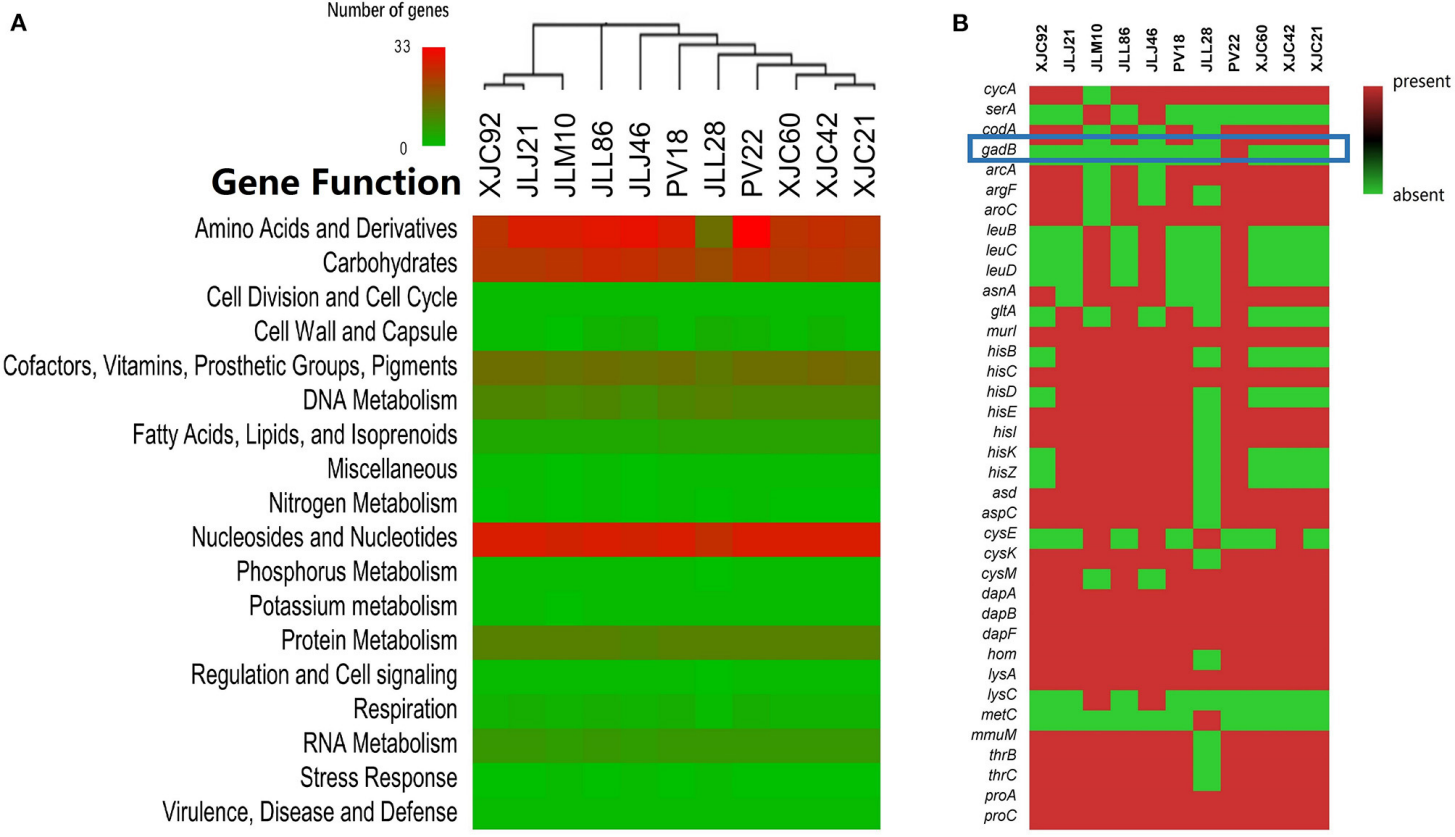
Genome mining of antiviral mechanisms of *Limosilactobacillus fermentum* PV22. **(A)** Gene distributions in different *L. fermentum* isolates. *L. fermentum* PV22 showed high abundance in gene processing, especially in amino acids and derivatives. **(B)** The presence/absence of genes that encode functional amino acids and derivatives. *gadB* is highlighted using a blue square in the heatmap; it is an *L. fermentum* PV22 strain-specific gene.

Pan-genome analyses revealed that *L. fermentum* PV22 shared 921 core genes (>99% presence) and 259 softcore genes (95% ≤ strain presence < 99%) with 77 *L. fermentum* strains from the NCBI database and ten strains isolated in our study. The present–absent status of protein-coding genes from the 11 isolated *L. fermentum* strains is shown in Figure 4. We identified 137 strain-specific genes in *L. fermentum* PV22, most of which encode transposase proteins. Among all genes encoding functional exocrine proteins, we only identified a strain-specific gene, *gadB*, which is reportedly associated with antiviral functions. Collectively, these results indicate that the strain-specific antiviral activity may be attributed to *gadB* in *L. fermentum* PV22.

#### Quantification of GABA in MNTDs of LABs

As *gadB* is a crucial gene for LAB to convert glutamate to GABA, we examined GABA levels to assess glutamate decarboxylase activity in MNTDs of LABs (Supplementary Table 3). The results of Triple Quad LC-MS/MS analysis showed that the GABA level in MNTDs of *L. fermentum* PV22 was 45.757 ± 0.315 mg/L, which was significantly elevated compared with that in the MRS broth (1.141 ± 0.003 mg/L) and that of other three *L. fermentum* isolates without *gadB* (1.572 ± 0.672 mg/L). This result showed that the MNTDs of *L. fermentum* PV22 contained high levels of GABA, which is an active antiviral compound.

#### Verification of the Antiviral Activity of GABA in MNTDs of LABs

We further analyzed the antiviral activity of GABA against MNV in RAW264.7 cells. Our results showed that pH-adjusted MRS with 44.67 mg/L of GABA reduced -log TCID_50_ of MNV titers to 2.998 ± 0.123, significantly lower than that measured without additional GABA (4.625 ± 0.125, *P* = 0.025). Our results indicate that GABA is an important antiviral metabolite of *L. fermentum* PV22. Notably, the antiviral effect was stronger in the MNTD of *L. fermentum* PV22 than MRS with GABA (–log TCID_50_ 2.542 ± 0.315 vs. 2.998 ± 0.123, *P* = 0.039), indicating there might be other antiviral metabolites in the MNTD.

#### Gene Transferability Analysis

**Figure 5:**
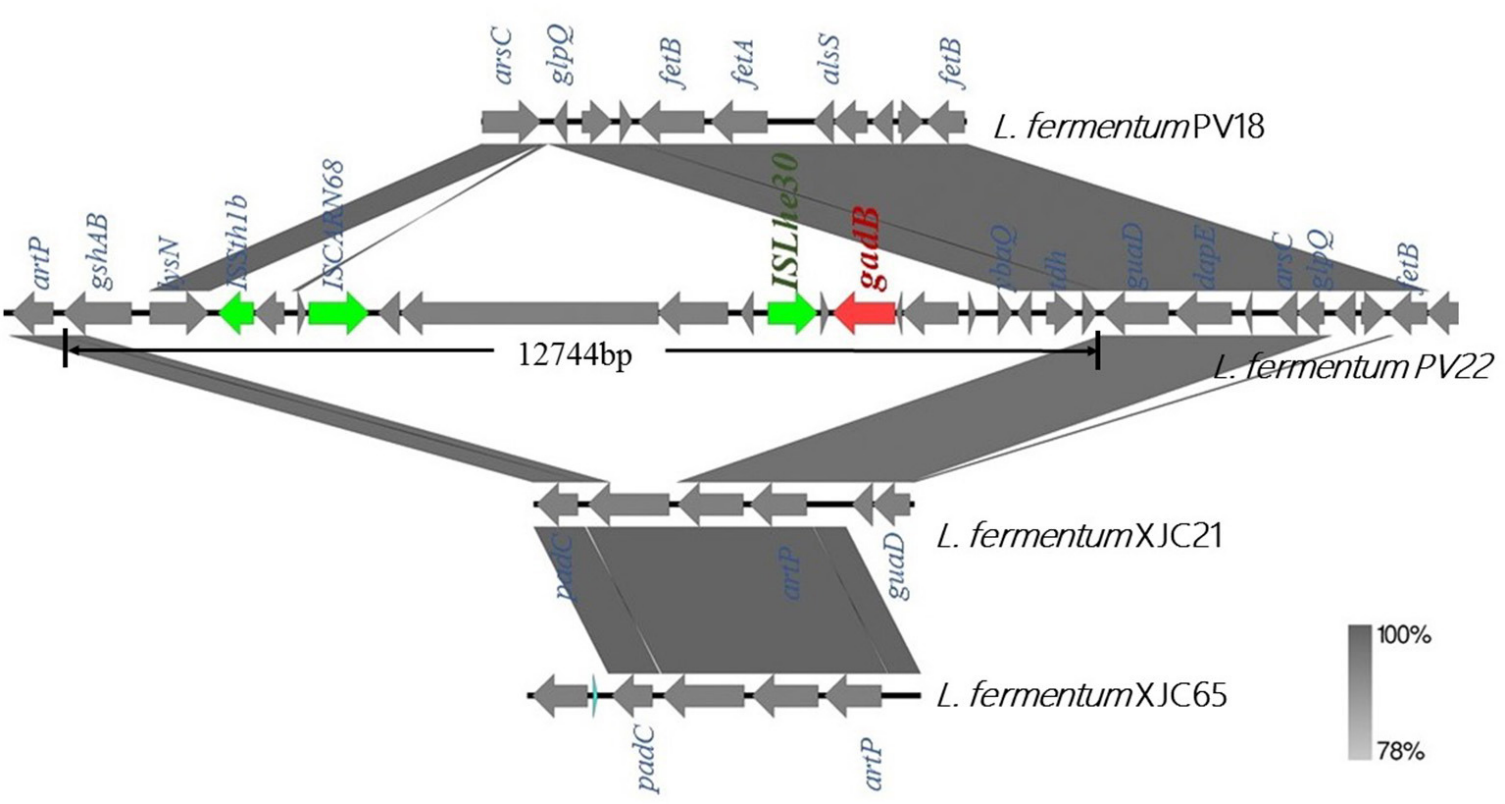
Genome analysis of *gadB*-expressing *Limosilactobacillus fermentum* PV22. Comparison of linear sequences derived from *L. fermentum* PV22 with those from other isolated strains of *L. fermentum*, including healthy centenarian gut-derived strain *L. fermentum* PV18 and traditional homemade yogurt-derived strains *L. fermentum* XJC21 and XJC65. *gadB* (red arrow) is located on an insertion sequence of 12,744 bp. Boxed arrows represent the position and transcriptional direction of open reading frames (ORFs). ORFs encoding transposases are marked in green.

Next, we compared the genomic organization of *L. fermentum* PV22, PV18, XJC21, and XJC65 (Figure 5). *L. fermentum* PV22 contains a 12,744-bp insertion sequence comprised of 20 protein-coding genes, of which ten encode proteins with functional assignments and ten encode hypothetical proteins. *gadB*, which encodes glutamate decarboxylase, was present in this inserted sequence, whereas the gene-encoding transposase *ISLhe30* was found upstream of *gadB*. The unique genomic structure of *L. fermentum* PV22 indicates that this specific strain may have acquired the ability to produce glutamate decarboxylase during the evolution of gut microbiome.

### Probiotic Potential of *L. fermentum* PV22

#### Safety Evaluation of *L. fermentum* PV22

No virulence or antibiotic-resistance genes were detected in the genome of *L. fermentum* PV22, as determined *via* a BLAST search of the VFDB, Resfinder, and CARD databases. In addition, *L. fermentum* PV22 exhibited no hemolysis on blood agar, further confirming the safety of the LAB strain (Supplementary Figure 2A). We also evaluated the antibiotic susceptibility of *L. fermentum* PV22 (Table 1) and concluded that *L. fermentum* PV22 is likely to be susceptible to all antibiotics required by EFSA.

**Table 1:** Antibiotic susceptibility of *Limosilactobacillus fermentum* PV22 to eight antibiotics tested in this study.

**Antibiotics**	**Minimum inhibitory concentration (mg/L)**	**Phenotype**
Ampicillin	0.5	Sensitive
Gentamicin	2	Sensitive
Kanamycin	32	Sensitive
Streptomycin	16	Sensitive
Erythromycin	0.5	Sensitive
Clindamycin	1	Sensitive
Tetracycline	2	Sensitive
Chloramphenicol	1	Sensitive

#### Gastrointestinal Tract Tolerance and Cell Adhesion Activity of *L. fermentum* PV22

When *L. fermentum* PV22 (1 × 10^8.2^ CFU/mL) was incubated with simulated gastric juice followed by simulated pancreatic juice, the cell count decreased to 1 × 10^7.7^ and 1 × 10^7.6^ CFU/mL, respectively, with a final survival rate in the gastrointestinal tract of 25.12% (Supplementary Figure 2B). Together, these results suggest that *L. fermentum* PV22 has a strong gastrointestinal tract tolerance. Moreover, an assessment of the cell adhesion activity revealed that 1 × 10^7.9^ CFU/mL cells of *L. fermentum* PV22 were attached to the Caco-2 cells, which suggests that 50.12% of the bacteria were attached to these cells, indicating excellent cell adhesion (Supplementary Figure 2C).

## Discussion

Human norovirus is the most common cause of foodborne gastroenteritis-related deaths and is responsible for 70,000 deaths in children under 5 years of age (2, 52, 53). Despite worldwide efforts to control the virus, progress has been slow and no effective treatment for norovirus exists to date (54, 55). Therefore, novel strategies to prevent norovirus contamination in food products and treat norovirus infections are urgently required.

In the food industry, fermentation can effectively reduce norovirus titers and prevents virus infections; studies have shown that certain LAB strains and their metabolites exhibit antiviral potential (56, 57). As LAB are the most commonly used probiotics and are widely consumed in our daily lives, they are ideal candidates for preventing and treating norovirus infection in the food industry and healthcare services (58). Until now, most studies on the antiviral activities of LAB strains against norovirus have been limited to food isolates, but increasing evidence suggests that LAB strains derived from the human gut and breast milk are highly tolerant to the gastrointestinal tract environment and are beneficial for the hosts (59). Therefore, human-isolated LABs are considered highly valuable strains in the healthcare industry, and more efforts should be directed toward exploring the probiotic potential of these strains (59). In this study, we included LABs from both traditional and unconventional sources, to broaden the scope for LAB screening.

In previous studies, the antiviral activity was only found in certain strains of LAB, and the active metabolites remained largely unexplored. To investigate the mechanism underlying the antiviral activity of LAB, we evaluated and compared the antiviral potential of 78 centenarian gut-derived strains and 44 fermented food-derived strains. Interestingly, we discovered that the gut-derived LAB strains conferred stronger protection to the virus-infected cells than the food-derived strains. Certain LAB species, such as *L. casei* and *L. rhamnosus*, displayed higher antiviral potential than other species, such as *L. plantarum*. Moreover, the gut-derived isolates exhibited superior deactivation activity among the same *Lactobacillus* species. Altogether, these results indicated that the LAB isolated from the human gut, especially the healthy centenarian gut, might have a better antiviral potential.

Based on the abovementioned findings, we further investigated whether the MNTDs of LAB could directly reduce the virus titers *in vitro* and whether they could be applied in the deactivation of virus in the food industry. Our results show that even though some LAB strains showed strong protective effects with regard to cell viability, they could seldom effectively reduce the virus titers. Among the most potential antiviral LAB strains, we identified a healthy centenarian gut-derived strain, *L. fermentum* strain PV22, which could significantly reduce the viral titer by 2.23 ± 0.38 log-value in a short co-incubation time. The antiviral activity of *L. fermentum* strain PV22 was even stronger than that of previously reported probiotic strains (60). Furthermore, we found that the antiviral activity of *L. fermentum* PV22 was stable and persistent at room temperature, indicating its great potential for applications in the food industry.

Although various clinical trials have demonstrated the antiviral effects of probiotics, only a few studies have explored the underlying mechanisms. Thus, the specific antiviral metabolites of LAB have not been clearly identified (2, 11, 27). Several studies have indicated that probiotics exert their effects by producing metabolites that may hinder the adsorption and internalization of viruses (61), directly kill viruses *in vitro* (14), and stimulate the antiviral immunity of the host (39). As most of these antiviral substances are present in the extracellular secretions of LAB (39), we performed a comparative analysis of genes that encode extracellular-secreted proteins to determine the potential molecular mechanism of the strongest antiviral strain *L. fermentum* PV22. Data mining revealed that compared to the strains without antiviral activities, *L. fermentum* PV22 harbored a unique gene, *gadB*, in its genome. Previous studies have revealed that *gadB* encodes glutamate decarboxylase B (62), the rate-limiting enzyme in GABA synthesis. GABA is an important antiviral molecule as it confers protection from a wide spectrum of viruses, ranging from herpes-zoster virus to influenza virus (63, 64). Researchers have proved that GABA can function as an immunological molecule and transmit signals *via* the “GABA shunt” pathway, thereby, modulating hypoxia-inducible factor 1α and interleukin-1β production (65, 66). Clinically, GABA prevents enterovirus infections (67) and relieves the symptoms of coronavirus infections and reduces mortality (60). Moreover, it has also reported that the glutamate decarboxylase protein has a structure highly similar to that of the coxsackievirus and enterovirus, and consumption of glutamate decarboxylase could evoke the immunological surveillance against these viruses and reduce the risk of virus infection (62). Our results prove that *L. fermentum* PV22 might synthesize high levels of GABA in the MNTD, which was associated with antiviral activities against MNV.

Furthermore, we analyzed the possible origins of *gadB* to elucidate the strain-specific antiviral effect of *L. fermentum* PV22. Linear sequence comparison between *L. fermentum* PV22 and other *L. fermentum* isolates revealed that *gadB* is present within a 12,744-bp insertion sequence, with the transposase-encoding gene *ISLhe30* located upstream. This unique structure in the *L. fermentum* PV22 genome indicates that PV22 may have acquired *gadB* under evolutionary pressures in the human gut and, thereby, developed the ability to synthesize GABA to modulate the gut microbiome. Together, the probable antiviral mechanisms of *L. fermentum* PV22 may be related to GABA generation, which was acquired owing to evolutionary pressures on the human gut and took effect by activation of the GABA shunt pathway in macrophages against the virus (66).

Safety and gastrointestinal tolerance are two important factors to consider when evaluating LAB strains as potential probiotics (68). In this study, we examined the safety of *L. fermentum* PV22 at both the genomic and physiological levels, with none of the tests suggesting any potential hazards. Moreover, simulated gastrointestinal tolerance tests showed that *L. fermentum* PV22 has good tolerance and excellent adhesion to the gastrointestinal tract. Therefore, healthy centenarian gut-derived *L. fermentum* PV22 demonstrates the potential for applications in the food and health care industries. However, further *in vivo* experiments should be performed to validate the antiviral effect of *L. fermentum* PV22 against norovirus.

## Conclusions

In this study, we identified a healthy centenarian gut-derived LAB strain, *L. fermentum* PV22, as a strong antagonist against MNV activity. We explored the molecular mechanisms operating in this gut-derived *L. fermentum* by comparative genome mining and concluded that the generation of glutamate decarboxylase, as well as GABA, may play an essential role in viral inhibition. Overall, our research provides a new understanding of the molecular mechanism of probiotics against viral infections. Moreover, these novel findings offer a solution for the prevention of norovirus infection and indicate the potential for the application of GABA-producing LAB strains in antiviral treatment.

## Data Availability Statement

The datasets presented in this study can be found in NCBI repositories. The name of the repository and accession numbers can be found below: NCBI; PRJNA703332, PRJNA703368, PRJNA703369, PRJNA703370, PRJNA703371, PRJNA703372, PRJNA703373, PRJNA703374, PRJNA703375, PRJNA703376.

## Author Contributions

YL, LX, YD, and QW designed the research study. YL, JG, and LX performed the antiviral research. YS, TJ, and HC contributed to the isolation, whole-genome sequencing, and probiotic evaluation of probiotics. YL, WC, and XX analyzed the data. YL and LX wrote the paper. JZ, JW, MC, and QW performed critical revisions of the manuscript. All authors contributed to the article and approved the submitted version.

## Funding

This research was funded by the National Natural Science Foundation of China (Grant Number 31872912), the Guangdong Provincial Key Laboratory (Grant Number 2020B121201009), the Natural Science Foundations of Guangdong Province for Distinguished Young Scholars (Grant Number 2019B151502065), the Key Research and Development Program of Guangdong Province (Grant Number 2020B111126007), and GDAS' Project of Science and Technology Development (Grant Number 2020GDASYL-20200104008). The funders had no role in the design of the study, collection, analyses, interpretation of data, writing of the manuscript, and decision to publish the results.

## Conflict of Interest

The authors declare that the research was conducted in the absence of any commercial or financial relationships that could be construed as a potential conflict of interest.

## Publisher's Note

All claims expressed in this article are solely those of the authors and do not necessarily represent those of their affiliated organizations, or those of the publisher, the editors and the reviewers. Any product that may be evaluated in this article, or claim that may be made by its manufacturer, is not guaranteed or endorsed by the publisher.

## Abbreviations

CARD: Comprehensive Antibiotic Research Database
CFS: cell-free serum
DMEM: Dulbecco's modified Eagle's medium
EFSA: European Food Safety Authority
GABA: γ-aminobutyric acid
LAB: lactic acid bacteria
MNTD: minimum non-toxic dilutions
MRS: Man Rogosa Sharpe
NLV: Norwalk-like virus
WGS: whole-genome sequencing.
