# Supplementary material for: Determination of Antiviral Mechanism of Centenarian Gut-Derived Limosilactobacillus fermentum Against Norovirus
Source: Front Nutr. 2022 Mar 28;9:812623. doi: 10.3389/fnut.2022.812623 (PMC8997286; doi:10.3389/fnut.2022.812623)
Supplement: Supplementary file 1 [file Data_Sheet_1.docx]

**Supplementary Material**

**Supplementary Table S1.** Taxonomy and isolation information of 122 *Lactobacillus* strains

| LAB strain | Taxonomy | Isolation location | Isolation source | |
| --- | --- | --- | --- | --- |
| JLD65 | *L. plantarum* | Guangdong | feces of healthy centenarian |  |
| JLD66 | *L. plantarum* | Guangdong | feces of healthy centenarian |  |
| JLE15 | *L. plantarum* | Guangdong | feces of healthy centenarian |  |
| JLE50 | *L. plantarum* | Guangdong | feces of healthy centenarian |  |
| JLE53 | *L. brevis* | Guangdong | feces of healthy centenarian |  |
| JLE88 | *L. plantarum* | Guangdong | feces of healthy centenarian |  |
| JLJ14 | *L. plantarum* | Guangdong | feces of healthy centenarian |  |
| JLJ16 | *L. fermentum* | Guangdong | feces of healthy centenarian |  |
| JLJ19 | *L. fermentum* | Guangdong | feces of healthy centenarian |  |
| JLJ21 | *L. fermentum* | Guangdong | feces of healthy centenarian |  |
| JLJ32 | *L. pantheris* | Guangdong | feces of healthy centenarian |  |
| JLJ42 | *L. fermentum* | Guangdong | feces of healthy centenarian |  |
| JLJ46 | *L. fermentum* | Guangdong | feces of healthy centenarian |  |
| JLK69 | *L. plantarum* | Guangdong | feces of healthy centenarian |  |
| JLL10 | *L. plantarum* | Guangdong | feces of healthy centenarian |  |
| JLL2 | *L. plantarum* | Guangdong | feces of healthy centenarian |  |
| JLL28 | *L. fermentum* | Guangdong | feces of healthy centenarian |  |
| JLL29 | *L. brevis* | Guangdong | feces of healthy centenarian |  |
| JLL31 | *L. plantarum* | Guangdong | feces of healthy centenarian |  |
| JLL43 | *L. fermentum* | Guangdong | feces of healthy centenarian |  |
| JLL45 | *L. fermentum* | Guangdong | feces of healthy centenarian |  |
| JLL61 | *L. fermentum* | Guangdong | feces of healthy centenarian |  |
| JLL8 | *L. plantarum* | Guangdong | feces of healthy centenarian |  |
| JLL81 | *L. plantarum* | Guangdong | feces of healthy centenarian |  |
| JLL85 | *L. plantarum* | Guangdong | feces of healthy centenarian |  |
| JLL86 | *L. fermentum* | Guangdong | feces of healthy centenarian |  |
| JLL88 | *L. plantarum* | Guangdong | feces of healthy centenarian |  |
| JLM10 | *L. fermentum* | Guangdong | feces of healthy centenarian |  |
| JLM2 | *L. plantarum* | Guangdong | feces of healthy centenarian |  |
| JLM37 | *L. plantarum* | Guangdong | feces of healthy centenarian |  |
| JLM4 | *L. plantarum* | Guangdong | feces of healthy centenarian |  |
| PV1 | *L. plantarum* | Guangdong | feces of healthy centenarian |  |
| PV11 | *L. fermentum* | Guangdong | feces of healthy centenarian |  |
| PV12 | *L. fermentum* | Guangdong | feces of healthy centenarian |  |
| PV13 | *L. fermentum* | Guangdong | feces of healthy centenarian |  |
| PV14 | *L. fermentum* | Guangdong | feces of healthy centenarian |  |
| PV15 | *L. fermentum* | Guangdong | feces of healthy centenarian |  |
| PV16 | *L. equi* | Guangdong | feces of healthy centenarian |  |
| PV18 | *L. fermentum* | Guangdong | feces of healthy centenarian |  |
| PV19 | *L. casei* | Guangdong | feces of healthy centenarian |  |
| PV2 | *L. plantarum* | Guangdong | feces of healthy centenarian |  |
| PV20 | *L. fermentum* | Guangdong | feces of healthy centenarian |  |
| PV21 | *L. fermentum* | Guangdong | feces of healthy centenarian |  |
| PV22 | *L. fermentum* | Guangdong | feces of healthy centenarian |  |
| PV23 | *L. delbrueckii* | Guangdong | feces of healthy centenarian |  |
| PV27 | *L. delbrueckii* | Guangdong | feces of healthy centenarian |  |
| PV28 | *L. reuteri* | Guangdong | feces of healthy centenarian |  |
| PV3 | *L. plantarum* | Guangdong | feces of healthy centenarian |  |
| PV30 | *L. oris* | Guangdong | feces of healthy centenarian |  |
| PV33 | *L. plantarum* | Guangdong | feces of healthy centenarian |  |
| PV35 | *L. salivarius* | Guangdong | feces of healthy centenarian |  |
| PV37 | *L. plantarum* | Guangdong | feces of healthy centenarian |  |
| PV38 | *L. salivarius* | Guangdong | feces of healthy centenarian |  |
| PV4 | *L. plantarum* | Guangdong | feces of healthy centenarian |  |
| PV44 | *L. salivarius* | Guangdong | feces of healthy centenarian |  |
| PV46 | *L. equi* | Guangdong | feces of healthy centenarian |  |
| PV47 | *L. rhamnosus* | Guangdong | feces of healthy centenarian |  |
| PV49 | *L. plantarum* | Guangdong | feces of healthy centenarian |  |
| PV5 | *L. plantarum* | Guangdong | feces of healthy centenarian |  |
| PV50 | *L. rhamnosus* | Guangdong | feces of healthy centenarian |  |
| PV54 | *L. rhamnosus* | Guangdong | feces of healthy centenarian |  |
| PV55 | *L. plantarum* | Guangdong | feces of healthy centenarian |  |
| PV6 | *L. delbrueckii* | Guangdong | feces of healthy centenarian |  |
| PV63 | *L. mucosae* | Guangdong | feces of healthy centenarian |  |
| PV65 | *L. casei* | Guangdong | feces of healthy centenarian |  |
| PV66 | *L. plantarum* | Guangdong | feces of healthy centenarian |  |
| PV68 | *L. mucosae* | Guangdong | feces of healthy centenarian |  |
| PV7 | *L. delbrueckii* | Guangdong | feces of healthy centenarian |  |
| PV70 | *L. helveticus* | Guangdong | feces of healthy centenarian |  |
| PV74 | *L. plantarum* | Guangdong | feces of healthy centenarian |  |
| PV78 | *L. brevis* | Guangdong | feces of healthy centenarian |  |
| PV8 | *L. delbrueckii* | Guangdong | feces of healthy centenarian |  |
| PV9 | *L. fermentum* | Guangdong | feces of healthy centenarian |  |
| PVE77 | *L. plantarum* | Guangdong | feces of healthy centenarian |  |
| PVE88 | *L. plantarum* | Guangdong | feces of healthy centenarian |  |
| PVH34 | *L. plantarum* | Guangdong | feces of healthy centenarian |  |
| PVH6 | *L. plantarum* | Guangdong | feces of healthy centenarian |  |
| PVH69 | *L. fermentum* | Guangdong | feces of healthy centenarian |  |
| XJA11 | *L. equi* | Xinjiang | yogurt |  |
| XJA12 | *L. salivarius* | Xinjiang | yogurt |  |
| XJA14 | *L. equi* | Xinjiang | yogurt |  |
| XJA2 | *L. equi* | Xinjiang | yogurt |  |
| XJC14 | *L. reuteri* | Xinjiang | milk granule |  |
| XJC2 | *L. mucosae* | Xinjiang | koumiss |  |
| XJC20 | *L. delbrueckii* | Xinjiang | milk granule |  |
| XJC21 | *L. fermentum* | Xinjiang | milk granule |  |
| XJC23 | *L. gallinarum* | Xinjiang | milk granule |  |
| XJC36 | *L. helveticus* | Xinjiang | milk granule |  |
| XJC42 | *L. fermentum* | Xinjiang | milk granule |  |
| XJC5 | *L. oris* | Xinjiang | koumiss |  |
| XJC56 | *L. fermentum* | Xinjiang | milk granule |  |
| XJC59 | *L. fermentum* | Xinjiang | koumiss |  |
| XJC60 | *L. fermentum* | Xinjiang | milk granule |  |
| XJC65 | *L. fermentum* | Xinjiang | yogurt |  |
| XJC67 | *L. plantarum* | Xinjiang | yogurt |  |
| XJC69 | *L. plantarum* | Xinjiang | milk lump |  |
| XJC70 | *L. delbrueckii* | Xinjiang | milk lump |  |
| XJC80 | *L. fermentum* | Xinjiang | milk lump |  |
| XJC9 | *L. plantarum* | Xinjiang | milk lump |  |
| XJC91 | *L. plantarum* | Xinjiang | milk lump |  |
| XJC92 | *L. fermentum* | Xinjiang | milk lump |  |
| XJG17 | *L. equi* | Xinjiang | yogurt |  |
| XJG18 | *L. plantarum* | Xinjiang | goat's milk |  |
| XJG21 | *L. equi* | Xinjiang | goat's milk |  |
| XJG33 | *L. reuteri* | Xinjiang | goat's milk |  |
| XJG49 | *L. plantarum* | Xinjiang | goat's milk |  |
| XJK20 | *L. equi* | Xinjiang | goat's milk |  |
| XJK21 | *L. gasseri* | Xinjiang | goat's milk |  |
| XJK49 | *L. plantarum* | Xinjiang | goat's milk |  |
| XJP1 | *L. fermentum* | Xinjiang | milk pimple |  |
| XJP13 | *L. delbrueckii* | Xinjiang | yogurt |  |
| XJP29 | *L. delbrueckii* | Xinjiang | milk pimple |  |
| XJP30 | *L. fermentum* | Xinjiang | milk pimple |  |
| XJP38 | *L. delbrueckii* | Xinjiang | milk pimple |  |
| XJP44 | *L. fermentum* | Xinjiang | milk pimple |  |
| XJP54 | *L. delbrueckii* | Xinjiang | milk pimple |  |
| XJP61 | *L. delbrueckii* | Xinjiang | milk pimple |  |
| XJP68 | *L. crustorum* | Xinjiang | mare's milk |  |
| XJP69 | *L. oris* | Xinjiang | mare's milk |  |
| XJP70 | *L. reuteri* | Xinjiang | mare's milk |  |
| XJP82 | *L. plantarum* | Xinjiang | yogurt |  |
| XJP87 | *L. oris* | Xinjiang | yogurt |  |

**Supplementary Table S2.** Genomic characteristics of 11 strains of *L. fermentum* in this study

| **Strain** | **XJC92** | **JLJ21** | **JLM10** | **JLL86** | **JLJ46** | **PV18** | **JLL28** | **PV22** | **XJC60** | **XJC42** | **XJC21** |
| --- | --- | --- | --- | --- | --- | --- | --- | --- | --- | --- | --- |
| **Genome Size (Mb)** | 1.91 | 2.07 | 2.05 | 2.04 | 2.08 | 2.07 | 2.08 | 2.19 | 1.97 | 1.97 | 1.97 |
| **GC content (%)** | 51.73 | 51.63 | 51.69 | 51.85 | 51.51 | 51.61 | 51.5 | 51.22 | 51.65 | 51.64 | 51.66 |
| **Number of CDS** | 2160 | 2200 | 2171 | 2142 | 2320 | 2214 | 2297 | 2278 | 2003 | 2026 | 2016 |
| **Number of tRNA** | 58 | 57 | 56 | 58 | 57 | 58 | 58 | 58 | 58 | 58 | 58 |
| **Number of Repeat Regions** | 12 | 56 | 20 | 9 | 22 | 56 | 30 | 170 | 229 | 232 | 228 |
| **Number of rRNA** | 7 | 10 | 12 | 13 | 10 | 10 | 9 | 15 | 15 | 15 | 15 |
| **Gene Number in Subsystem** | | | | | | | | | | | |
| Protein Processing | 313 | 368 | 378 | 367 | 392 | 368 | 357 | 365 | 317 | 317 | 315 |
| Stress Response, Defense, Virulence | 194 | 194 | 194 | 194 | 195 | 194 | 194 | 194 | 185 | 185 | 185 |
| DNA processing | 67 | 71 | 73 | 77 | 70 | 70 | 69 | 79 | 75 | 75 | 73 |
| Energy | 80 | 79 | 84 | 78 | 84 | 79 | 78 | 81 | 81 | 81 | 81 |
| RNA processing | 96 | 86 | 92 | 93 | 102 | 92 | 89 | 83 | 98 | 98 | 96 |
| Cellular Processes | 40 | 40 | 40 | 40 | 41 | 40 | 40 | 40 | 40 | 40 | 40 |
| Regulation and Cell Signaling | 55 | 58 | 56 | 56 | 57 | 58 | 54 | 57 | 57 | 57 | 57 |
| Membrane Transport | 7 | 15 | 15 | 15 | 15 | 15 | 15 | 15 | 8 | 8 | 8 |

**Supplementary Table S3.** Quantification of γ-aminobutyric acid in the MNTDs of the *L. fermentum* isolates

| **Strain** | **γ-aminobutyric acid (mg/L)** |
| --- | --- |
| PV22 | 45.757 ± 0.315 |
| PV18 | 1.572 ± 0.672 |
| XJC21 | 2.774 ± 0.149 |
| XJC65 | 2.552 ± 0.096 |
| pH adjusted MRS | 1.141 ± 0.003 |

**Supplementary Figure 1**


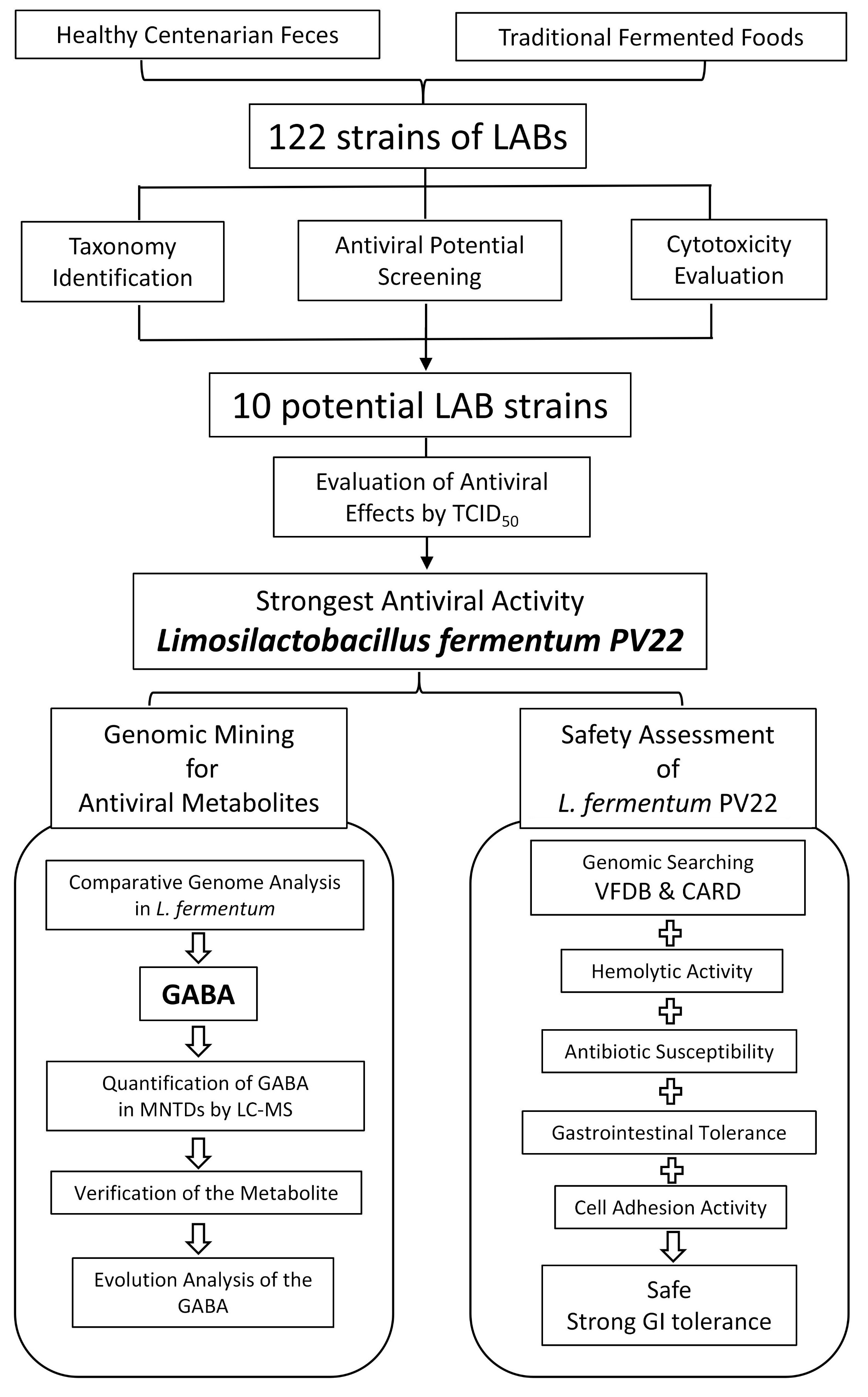


**Supplementary Figure 1 The flow diagram for the selection and verification of the lactic acid bacteria (LAB) strains with anti-norovirus activities**

LAB: lactic acid bacteria; TCID_50_: 50% tissue culture infectious dose; GABA: γ-aminobutyric acid; *L. fermentum*: *Limosilactobacillus fermentum*; MNTD: Minimum non-toxic dilutions; LC-MS: liquid chromatography-tandem mass spectrometry; VFDB: Virulence Factor Database; CARD: Comprehensive Antibiotic Research Database; GI: gastrointestinal.

**Supplementary Figure 2**

*
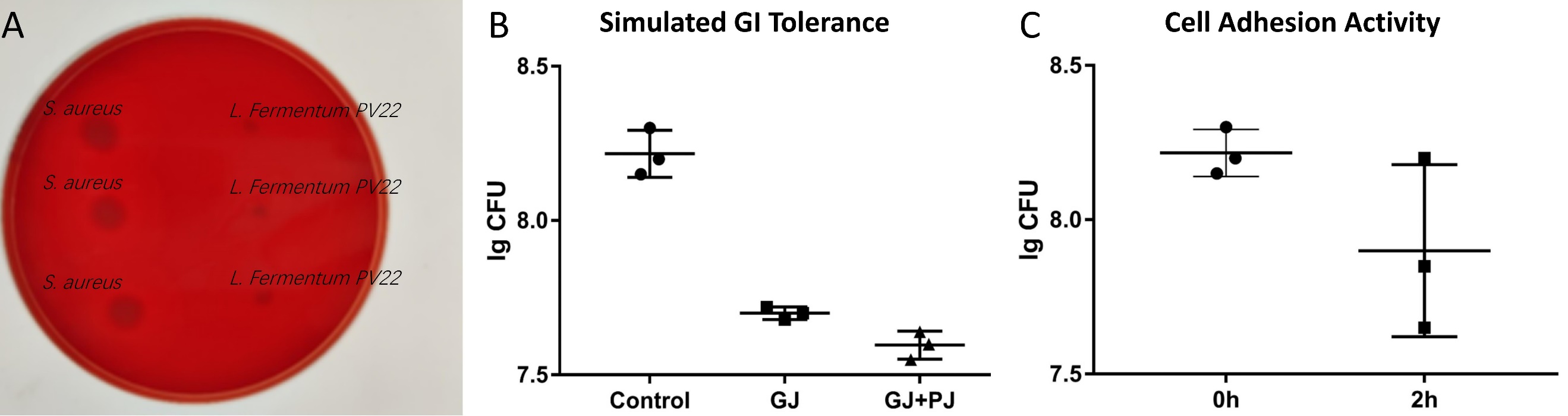
*

**Supplementary Figure 2 Safety and gastrointestinal tract tolerance assessment of *L. fermentum* PV22**

A. Hemolytic activity examination of *L. fermentum* PV22: compared to the positive control, no hemolytic phenomenon could be observed in *L. fermentum* PV22. B. Gastrointestinal tract tolerance of *L. fermentum* PV22: *L. fermentum* PV22 showed strong gastrointestinal tract tolerance against simulated gastric juice and simulated pancreatic juice. C. Cell adhesion activity of *L. fermentum* PV22: 50.12% of the bacteria were attached to the Caco-2 cells after 2 hours co-incubation.

*L. fermentum*: *Limosilactobacillus fermentum*; GI: gastrointestinal; GJ: gastric juice; PJ: pancreatic juice.
